# The Role of Resorbable Plate and Artificial Bone Substitute in Reconstruction of Large Orbital Floor Defect

**DOI:** 10.1155/2016/1358312

**Published:** 2016-07-19

**Authors:** Ho Kwon, Ho Jun Kim, Bommie F. Seo, Yeon Jin Jeong, Sung-No Jung, Hyung-Sup Shim

**Affiliations:** Department of Plastic and Reconstructive Surgery, College of Medicine, The Catholic University of Korea, Seoul 06591, Republic of Korea

## Abstract

It is essential to reduce and reconstruct bony defects adequately in large orbital floor fracture and defect. Among many reconstructive methods, alloplastic materials have attracted attention because of their safety and ease of use. We have used resorbable plates combined with artificial bone substitutes in large orbital floor defect reconstructions and have evaluated their long-term reliability compared with porous polyethylene plate. A total of 147 patients with traumatic orbital floor fracture were included in the study. Surgical results were evaluated by clinical evaluations, exophthalmometry, and computed tomography at least 12 months postoperatively. Both orbital floor height discrepancy and orbital volume change were calculated and compared with preoperative CT findings. The average volume discrepancy and vertical height discrepancies were not different between two groups. Also, exophthalmometric measurements were not significantly different between the two groups. No significant postoperative complication including permanent diplopia, proptosis, and enophthalmos was noted. Use of a resorbable plate with an artificial bone substitute to repair orbital floor defects larger than 2.5 cm^2^ in size yielded long-lasting, effective reconstruction without significant complications. We therefore propose our approach as an effective alternative method for large orbital floor reconstructions.

## 1. Introduction

The orbit is a four-sided pyramidal structure that comprises the roof, floor, and medial and lateral bony walls. A “blow-out fracture” is defined as a fracture that involves the orbital walls, especially the medial wall and/or orbital floor. Blow-out fractures constitute 22% to 47% of orbital injuries, as well as a large portion of midfacial fractures [1–5].

Orbital wall fractures, especially those that involve the orbital floor, are associated with complications such as diplopia from extraocular muscle entrapment, ecchymosis, eyelid edema, subconjunctival hemorrhage, and V2 sensory nerve deficit. Moreover, lack of sufficient treatment may lead to persistent enophthalmos and/or hypoglobus; therefore, it is essential to reduce and reconstruct bony defects adequately [1, 2, 6].

Many orbital floor reconstruction methods using various materials such as biological substances (autologous bone and cartilage grafts, bone and dural allografts, porcine collagen, and dermal xenografts), resorbable plates (poly l-lactic acid, polyglycolic acid, polydioxanone, composite polymers, and polycaprolactone), permanent plates (porous polyethylene, titanium mesh implant, and titanium mesh coated with porous polyethylene), or other alloplastic materials (silicone sheet and Teflon implant) have been developed to date [7–11].

All these methods are currently being widely used to reconstruct orbital floor defects, with a recent focus on alloplastic materials because of their safety and ease of use. A generalized consensus already exists regarding which materials are ideal for defects larger than 2.5 cm^2^ in size; nonetheless, certain disadvantages that result from the nonresorbable nature of these materials may arise [1, 5–9]. To overcome these aspects, we have utilized resorbable plates combined with artificial bone substitutes for large orbital floor defect reconstructions and have compared them with porous polyethylene, which is the most widely used permanent material in large orbital floor defect surgeries. We hereby present our findings together with relevant long-term follow-up data.

## 2. Patients and Methods

### 2.1. Ethical Statement

This study was approved by the Institutional Review Board of the Catholic University of Korea. All data was analyzed anonymously and according to the principles in the Declaration of Helsinki (1975, revised in 2008).

### 2.2. Patients

A total of 147 patients with traumatic orbital floor fracture were included in the study. All patients had orbital floor defects larger than 2.5 cm^2^, confirmed based on preoperative computed tomography (CT) scans, and received unilateral orbital floor reconstruction with either porous polyethylene plates or resorbable plates from January 2011 to January 2016 at our institute. The causes of injury included assault (78 patients, 53%), traffic accident (25 patients, 17%), sports accident (16 patients, 11%), industrial accident (14 patients, 10%), and fall (14 patients, 10%).

The reconstructive material used in each surgery was randomly assigned. Patients reconstructed with porous polyethylene plates were designated as the “control” group, while those reconstructed with resorbable plates and artificial bone substitutes were termed as the “combined” group. Exclusion criteria were as follows: bilateral orbital floor fractures, fractures involving other orbital walls, coexisting orbital rim fractures, patients with previous surgical history involving orbital walls, and any comorbidities that could hinder bone healing.

### 2.3. Description of Reconstructive Materials

#### 2.3.1. Resorbable Poly-L-Lactic Acid-Polyglycolic Acid (PLLA) Implant: LactoSorb®

LactoSorb (Biomet Microfixation, Jacksonville, FL, USA), a resorbable PLLA-PGA orbital implant, is available in both mesh and nonmesh plate forms. PLLA-PGA plates have already been proven to be effective when used alone in orbital floor reconstruction and are known to completely hydrolyze after 12 months [12–15]. In this study, we used a nonmesh, left- or right-sided anatomical type plate with a thickness of 0.25 mm.

#### 2.3.2. Artificial Bone Substitute: PolyBone®

PolyBone (Kyungwon Medical, Seoul, Korea) is a self-setting, calcium phosphate cement intended for use in the repair of craniofacial bone defects as well as in the augmentation or restoration of bony contours. PolyBone consists of beta-tricalcium phosphate, monocalcium monobasic, calcium sulfate hemihydrate, and polyphosphate. It is the only commercially available artificial bone product that contains polyphosphate to replace damaged bone in addition to promoting bone growth and was approved by the FDA in 2007 and the European CE in 2005 [16].

### 2.4. Surgical Procedure

**Figure 1:**
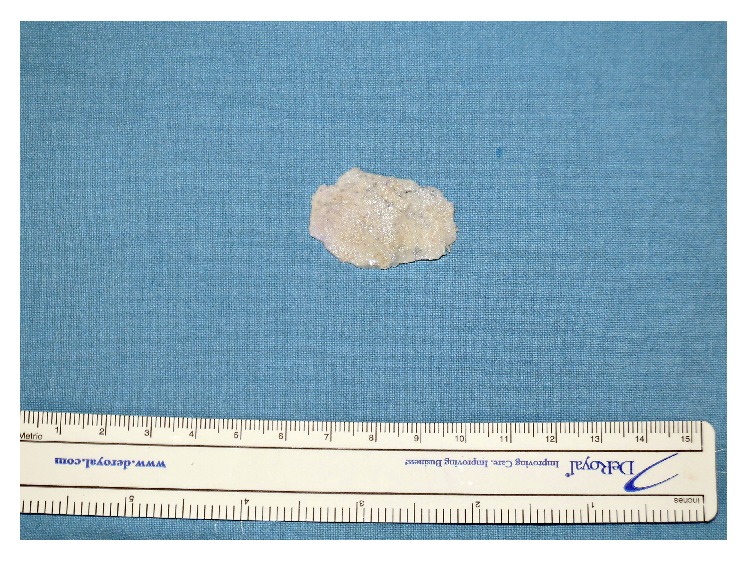
PolyBone paste formed by mixing and compressing PolyBone with fibrin glue. PolyBone paste was further trimmed and attached to the undersurface of a scored resorbable plate to just fit the area of the bone defect.

**Figure 2:**
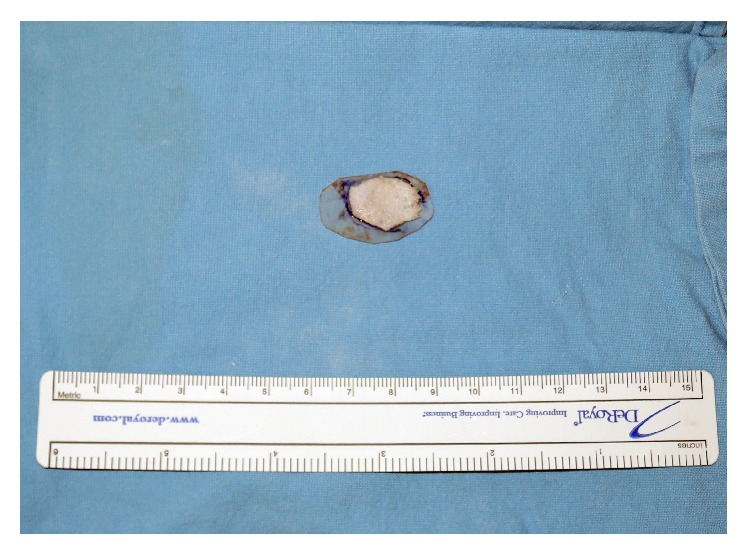
After the anatomical resorbable plate was trimmed to be slightly larger than the defect and scored undersurface, the PolyBone paste was attached to the scored undersurface of the plate with additional fibrin glue.

**Figure 3:**
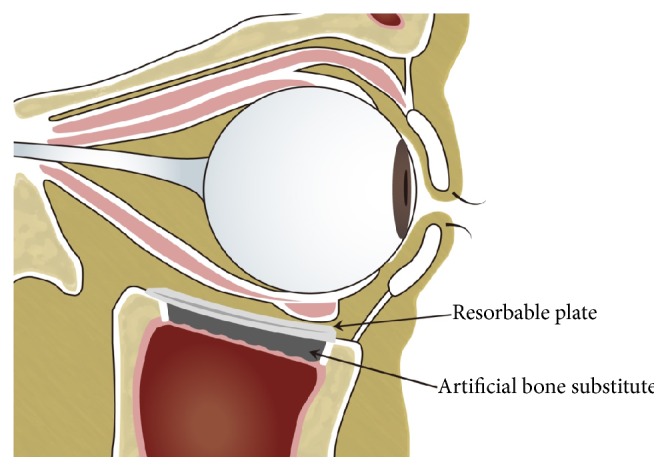
Schematic diagram after setting of the resorbable plate combined with the artificial bone substitute onto the bone defect area of the orbital floor.

Under general endotracheal anesthesia, a subciliary approach was taken to expose the infraorbital rim. Following a periosteal incision 2-3 mm below the infraorbital margin, the orbital floor defect was exposed through subperiosteal dissection. All the nonvitalized, displaced fractured segments were carefully removed while being cautious not to cause maxillary mucosal injury. The porous polyethylene or resorbable plate was prepared by trimming it to be slightly larger than the actual defect size. Additionally, in the “combined” group, multiple scoring with a No. 15 blade was performed to etch the inferior aspect of the resorbable plate, and a “PolyBone paste” formed by mixing PolyBone and fibrin glue (Figure 1) was then applied over the plate to match the bone defect (Figures 2 and 3), with the multiple grooves acting to secure the paste to the plate.

The prepared plate was then inserted over the defect, and the infraorbital periosteum was repaired with absorbable sutures to minimize unwanted plate displacement. Forced duction test was performed to confirm no extraocular muscle or soft tissue entrapment, followed by skin closure.

### 2.5. Evaluation of Reconstruction Results

**Figure 4:**
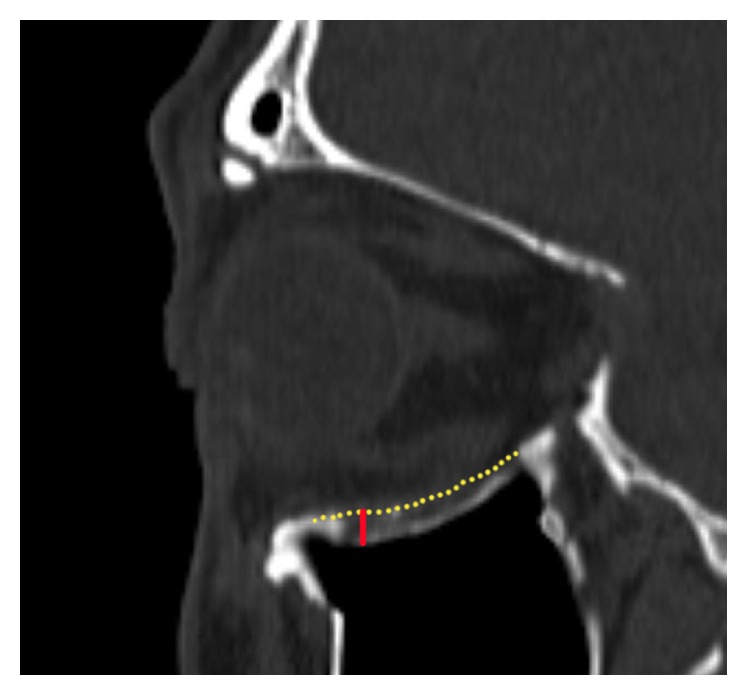
Sagittal view of the CT from the 14-month follow-up of a patient reconstructed with resorbable plate only is shown to depict how the postoperative results were evaluated. The dotted yellow line depicts the normal baseline orbital floor inferred from the contralateral noninjured orbit, and the red line depicts the greatest height discrepancy between the imaginary normal yellow dotted line and the actual reconstructed orbital floor. The area between the yellow line and actual orbital floor was integrated to calculate the increase in orbital volume after surgery.

Pre- and postoperative symptoms related to the orbital wall fracture including diplopia, pain and discomfort during eyeball movement, binocular visual acuity, and gross periorbital asymmetry were documented. All patients underwent preoperative 3D CT imaging to determine bone defect sizes obtained at a thickness of 1 mm for accurate area and volume calculations. Postoperative follow-up CT scans were performed at least 12 months after surgery, because it takes 12 months for complete hydrolysis of LactoSorb. Estimated initial anatomical dimensions of the affected orbital floor were created based on the patient's uninjured side, and the resultant imaginary orbital floor was set as baseline for calculating the highest difference in vertical position of the orbital floor and increased orbital volume from postoperative CT images (Figure 4). Increased orbital volumes were analyzed by integrating the increased areas obtained from sagittal CT scan imaging, as described in previous studies [17–22]. Also, pre- and postoperative clinical photography and exophthalmometric measurements were recorded in all cases.

## 3. Results

Statistical analyses were conducted using SAS software version 9.3 (SAS Institute, Cary, NC, USA) with an independent sample *t*-test, and *p* < 0.05 was considered significant. Patient demographic data are presented in Table 1.

A total of 147 patients were included in the study, with 52 patients in the control group (38 males and 14 females) and 95 patients in the combined group (68 males and 27 females). The mean age of the control group was 44 (range 19–69) years, while that of the combined group was 42 (range 18–69) years; this age difference was not statistically significant. Average follow-up duration of the control group was 23 (range 16–34) months, compared to 27 months for the combined group (range 17–37 months).

**Figure 5:**
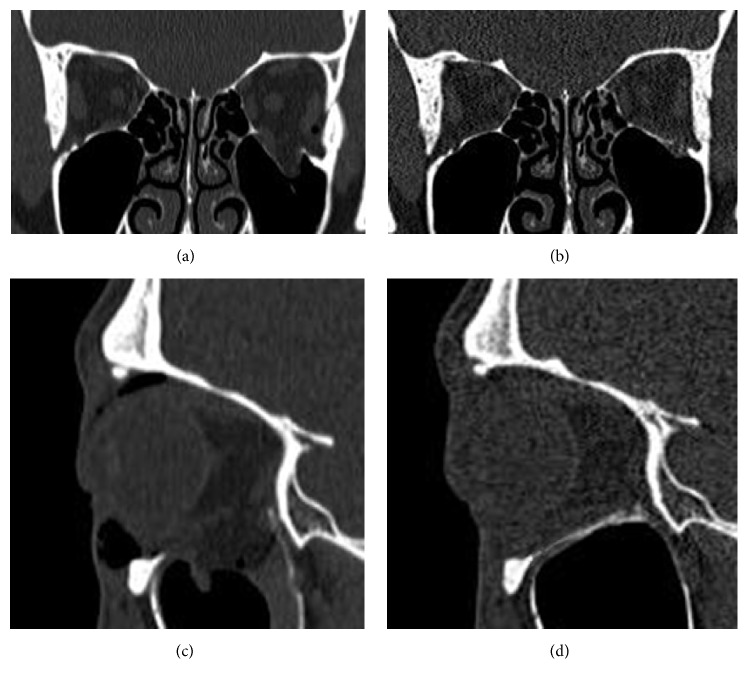
Pre- and postoperative CT views of a 38-year-old male patient who had a large orbital defect reconstructed with a resorbable plate and artificial bone substitute. Coronal view of preoperative CT showed a left-sided orbital floor defect of 4.2 cm^2^ with inferior rectus muscle displacement (a). This patient had discomfort with upper gaze, but no extraocular muscle limitation. Eighteen-month postoperative CT scan (coronal view) showed a reconstructed orbital floor with thin bone compared with the opposite side (b). The sagittal plane of the pre- and postoperative CT images revealed a nearly anatomically reconstructed orbital floor with no orbital volume discrepancy (c, d).

The average area size of the orbital floor defect obtained from preoperative CT images was not significantly different between the control and combined groups at 3.24 ± 0.61 cm^2^ (mean ± SD) and 3.11 ± 0.53 cm^2^ (mean ± SD), respectively. Also, average volume discrepancies between the estimated initial orbital cavity and actual values obtained from postoperative CT images in the control and combined group were 14.1 ± 8.2 mm^3^ (mean ± SD) and 12.7 ± 4.9 mm^3^  (mean ± SD), respectively (Figure 5), showing no significant difference. Furthermore, vertical height discrepancies between the estimated initial vertical height and actual measured floor height were similar between the two groups at 0.13 ± 0.09 mm in the control group (mean ± SD) and 0.15 ± 0.12 mm in the combined group (mean ± SD). These objective data suggest that the postoperative results achieved in the combined group were as reliable as the control group that utilized the standard reconstructive method (Table 2).

Postoperative exophthalmometric measurements were obtained at least 12 months after the surgery, and the average discrepancy between the affected and normal eye in the control group was 0.3 ± 0.1 mm (mean ± SD) versus 0.2 ± 0.1 mm (mean ± SD) in the combined group, which was not a statistically significant difference.

**Figure 6:**
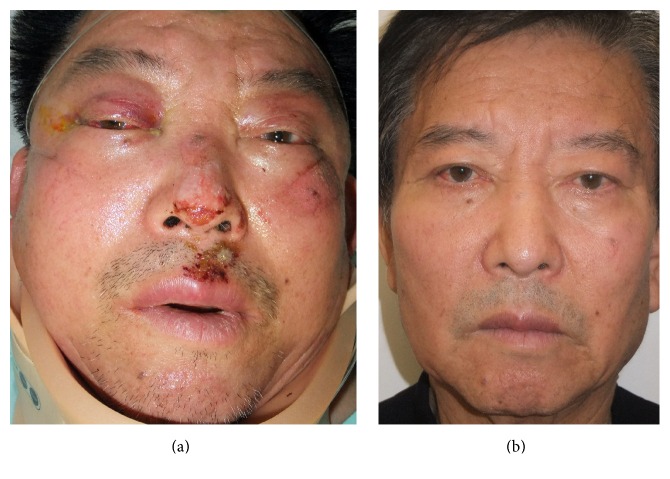
Pre- and postoperative clinical photographs of a 51-year-old male patient underwent a reconstruction of the right-sided orbital floor defect with a resorbable plate combined with artificial bone substitute. (a) Preoperative photograph shows panfacial swelling with multiple abrasion and conjunctival ecchymosis. (b) Postoperative 12-month photograph. No visible scar on subciliary incision site and no vertical overcorrection at neutral gaze are noted.

Neither group presented with any immediate postsurgical vertical overcorrections that could result from using a thick implant (Figure 6). Other notable complications such as seroma, chronic granuloma, exophthalmos, mucosal hypertrophy, and chronic infectious state did not arise; however, eight patients complained of temporary diplopia which resolved spontaneously within 1 month, and three patients experienced subcutaneous hematoma resolved with simple drainage. Five patients showed permanent decrease in visual acuity, a symptom that had already existed preoperatively. The rest of the patients were discharged without further complications and did not exhibit any late complications such as delayed enophthalmos, periorbital asymmetry, proptosis, or diplopia.

## 4. Discussion

Resorbable plates are effective in orbital floor reconstruction for defects smaller than 2.5 cm^2^ because they support the orbital floor with fibrous connective tissue through hydrolysis and promote osteoinduction. However, debate still remains about their efficacy in managing larger bone defects. Even after plate insertion, late complications such as enophthalmos may arise in cases in which comminuted bone fragments have already been devitalized or in cases where the plate fails to provide sufficient osteoinduction capacity. Nevertheless, there have been reports of successful large defect reconstructions using resorbable plates only, where surgically repaired anatomical structures were still intact long after the initial operation [12–15]. This may be due to the constant pressure applied on the orbital cavity by the maxillary sinus, which acts as a reduction force that ultimately facilitates fractured bone healing.

Many alternative methods to repair large orbital floor defects have been proposed, including approaches that use autologous materials such as bone and cartilage, or alloplastic, nonresorbable materials such as porous polyethylene and titanium mesh [1, 5, 8–11].

Although autologous material and porous polyethylene plate with/without titanium mesh have been utilized as ideal reconstructive material for large orbital floor defect, autologous materials inherently have many drawbacks including donor site morbidity, limited availability, and patient refusal. Moreover, the permanent polyethylene plate has disadvantages such as potential risk of infection, extrusion, and nonmalleability. These limitations have urged the development alternative alloplastic materials in orbital floor reconstruction, with a recent focus on resorbable plates in particular because of their anatomical form, ease of molding, and hydrolyzing properties.

In an effort to utilize these characteristics in large orbital floor defect reconstructions, we decided to simultaneously use an artificial bone substitute and a resorbable plate. Various artificial bone substitutes have been developed to date and are used in craniofacial bone surgeries for their safety and stability.

Prior to this study, Sakamoto et al., reported using a 0.5 mm thick resorbable mesh plate and bone cement (calcium phosphate cement, Biopex; HOYA Corporation, Tokyo, Japan) to reconstruct the orbital floor [23]. However, their approach differed from ours in that they covered the mesh plate with bone cement, resulting in thicker, nonanatomical bone formation that could potentially lead to exophthalmos.

In our study, we used a 0.25 mm thick anatomical resorbable plate to facilitate survival of the artificial bone substitute and minimized the infection rate by fixating the material to the inferior aspect of the plate. Both acute and chronic infection rate in study group were zero, which proves minimal risk of infection. The resulting thin bone formation in turn led to a more physiological repair. Another advantage of using a thin anatomical resorbable plate with an artificial bone substitute is that it can recreate the concavity and convexity of a normal globe, providing adequate protection should the patient experience another traumatic event. Moreover, using a thin plate can prevent possible exophthalmos that can occur when using another thick alloplastic plate. We utilized PolyBone as an artificial bone substitute. PolyBone is a mixture of calcium phosphate and polyphosphate that has superior bone regeneration capacity to bone cement. It also releases very little thermal energy during chemical reaction so that any needless deformation of the preformed resorbable plate is obviated.

Orbital volume measurement is one of the tools that can be used to evaluate the result of a successful orbital fracture reconstruction. A discrepancy of 50 mm^3^ or more between the orbital volume dimensions of a patient generally translates into a difference of 1 mm or more on exophthalmometric measurements [22]. Based on the given assumption, if a volume difference greater than 50 mm^3^ had been created after the surgery, the patient would have been clinically diagnosed with enophthalmos. Although there were no cases of clinically detected enophthalmos in either group except for one patient in the control group, there was significantly less difference between the postsurgical and estimated initial values of both the orbital volume and floor height in the combined group than the control group, suggesting that combining an artificial bone substitute with a resorbable plate yields superior results to those that can be achieved using a resorbable plate only.

Because we utilized thin, anatomical resorbable plates as opposed to thick, meshed plates, complications such as temporary vertical overcorrection, which frequently arise when using thick alloplastic materials, were avoided. Moreover, rates of immediate postoperative discomfort and temporary diplopia were low.

A major limitation of this study is that the technique presented above may not be easily applied for larger defects that involve medial or lateral orbital walls. Nonetheless, other reconstructive materials can be used in these cases. Of note, fixating PolyBone to the plate may extend the operating time by few minutes, but this delay can be minimized with the help of a trained assistant.

Although not discussed in this study, we have also used our reconstructive method to repair other orbital wall defects with concomitant maxillofacial fractures and were able to achieve sufficient bone defect reconstruction; this will be reported in a future study.

## 5. Conclusion

We used resorbable plates with artificial bone substitute rather than porous polyethylene plates only to repair orbital floor defects larger than 2.5 cm^2^ and obtained long-lasting, effective reconstruction with minimal complications. Based on these results from the first large scale study, we recommend using the resorbable plate with artificial bone substitute as a good alternative technique for large orbital floor reconstructions.

## Figures and Tables

**Table 1:** Demographic data of patients included in the study.

	Control group	Combined group
Total	52	95
Male	38	68
Female	14	27

Age (years)	44 (range 19~69)	42 (range 18~69)

Follow-up (months)	23 (range 16~34)	27 (range 17~37)

Defect size (cm^2^, mean ± SD)	3.24 ± 0.61	3.11 ± 0.53

**Table 2:** Postoperative volume discrepancy, vertical height discrepancy, and exophthalmometry discrepancy of two groups.

	Control group	Combined group
Volume discrepancy (mean ± SD)	14.1 ± 8.2 mm^3^	12.7 ± 4.9 mm^3^
Vertical height discrepancy (mean ± SD)	0.13 ± 0.09 mm	0.15 ± 0.12 mm
Exophthalmometry discrepancy (mean ± SD)	0.3 ± 0.1 mm	0.2 ± 0.1 mm
